# Radiation therapy for amyloid‐producing odontogenic tumor in a cat: a case report

**DOI:** 10.1111/avj.70021

**Published:** 2025-09-13

**Authors:** A Uno, T Mori

**Affiliations:** ^1^ Animal Medical Center, Gifu University Gifu Japan; ^2^ Department of Veterinary Clinical Sciences Gifu University Gifu Japan

**Keywords:** amyloid‐producing odontogenic tumor, cat, odontogenic tumor, radiation therapy

## Abstract

Amyloid‐producing odontogenic tumor (APOT) is a rare odontogenic neoplasm in cats, characterized by amyloid deposition within the tumor. Surgical resection is commonly recommended, but in cases where complete excision is difficult, radiation therapy may be considered as an alternative treatment. In this case report, a 10‐year‐old male neutered domestic cat with an APOT of the maxilla was treated with radiation therapy and showed favorable outcomes. The treatment protocol involved a total dose of 42 Gy (Gray) administered over six sessions, with good tumor control, and minimal side effects observed over a 481‐day follow‐up period. This case suggests that radiation therapy can be an effective treatment option for APOT, particularly in cases where surgical excision is not feasible.

Amyloid‐producing odontogenic tumors (APOT) are rare odontogenic neoplasms in cats, characterized by amyloid deposition within the tumor mass. The presence of amelogenin as an amyloid‐associated protein has been detected in the amyloid deposits of APOTs in cats. While the role of odontogenic amyloid ameloblast‐associated protein (ODAM), which is found in human cases of calcifying epithelial odontogenic tumors, has not been clearly established in feline and canine APOTs, they are generally categorized as distinct neoplasms. These tumors can affect any part of the dental arcades, can be affected and usually present as nonmetastatic, solitary lesions. Although surgical resection is the main treatment recommended, because it has a high degree of local invasiveness and infiltrates the bone, complete resection may be difficult depending on the location and size of the tumor, and local recurrence may be observed. Radiation therapy may be considered in these instances, but its role remains undefined in the treatment of APOT. Although previous studies have described the pathological features of APOT in dogs and cats and recurrence after surgical resection, no established treatments other than surgery have been reported for cats.^1^

This report aimed to describe the case of a cat diagnosed with APOT that underwent radiation therapy with favorable results.

## Case report

**Figure 1:**
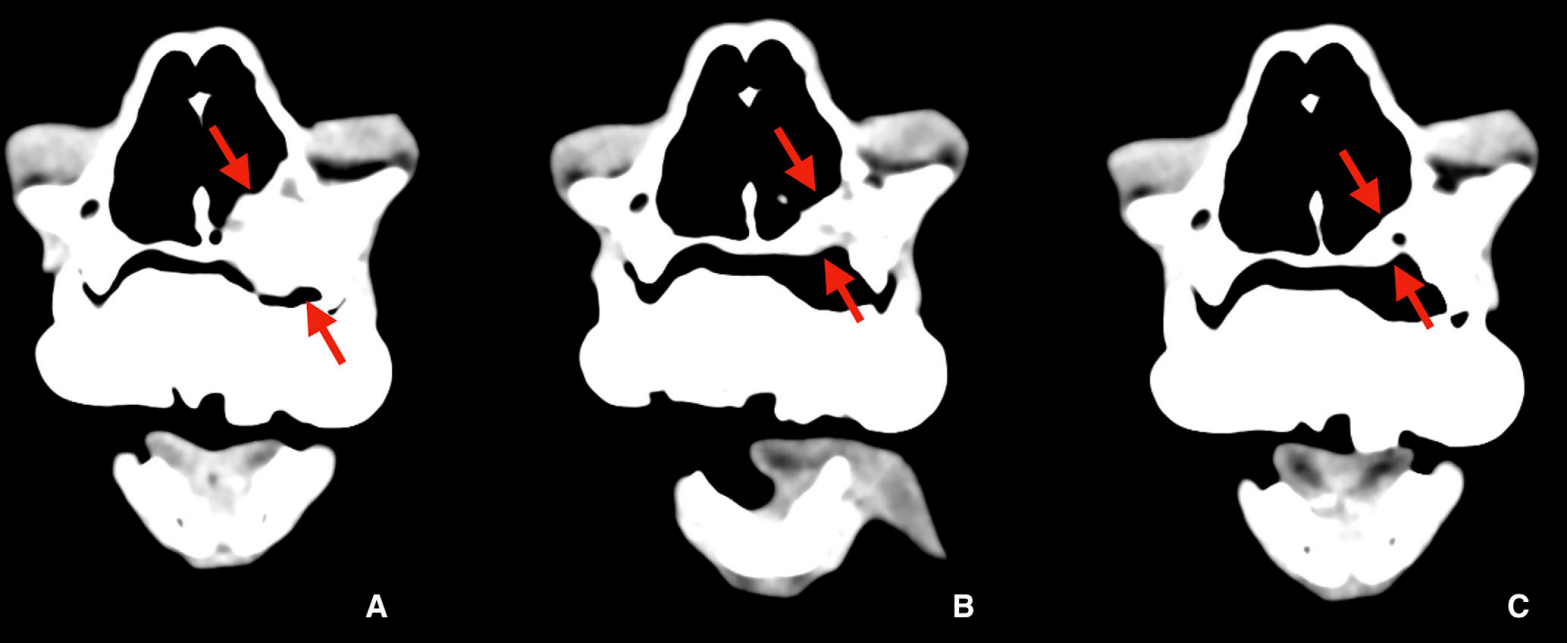
Contrast‐enhanced CT images of the maxilla of a cat with amyloid‐producing odontogenic tumor (APOT). (A) Pretreatment, (B) 2 months posttreatment, (C) 9 months posttreatment. The lesion is indicated with red arrows. (A) Pretreatment CT scan showing the mass (1.9 cm in length) with bone destruction and infiltration into the nasal cavity. (B) Two months posttreatment CT scan showing significant reduction in tumor size (1.1 cm). (C) Nine months posttreatment CT scan showing complete resolution of the tumor, with no recurrence observed.

**Figure 2:**
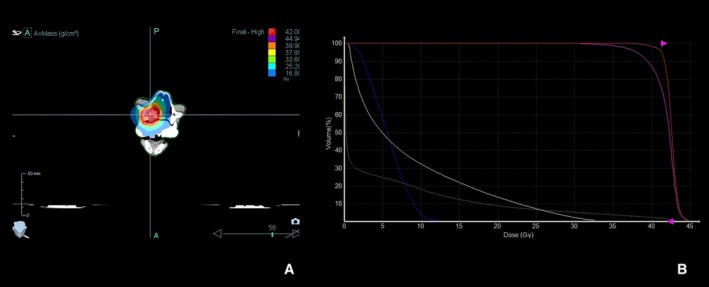
Radiation treatment plan for a cat with amyloid‐producing odontogenic tumor (APOT). (A) Dose distribution with isodose lines displayed on the treatment planning CT image. (B) Dose–volume histogram (DVH) showing coverage of the gross tumor volume (GTV, red line) and planning target volume (PTV, pink line), as well as doses delivered to surrounding tissues including external contour (green line), right eye (blue line), and left eye (yellow line). The DVH demonstrates adequate target coverage and acceptable doses to the eyes.

A 10‐year‐old, 6.4 kg male neutered domestic cat was presented to our clinic with anorexia and a mass lesion in the maxillary gingiva. Blood tests were unremarkable. Staging CT scans revealed a mass (1.9 cm in length) infiltrating the left maxilla with bone destruction and extension into the nasal cavity (Figure 1). Histopathology confirmed the diagnosis of APOT. Due to the owner's preference, surgical resection was not pursued, and radiation therapy was elected. Radiation treatment was delivered using the Accuray TomoTherapy HDA system. The treatment plan was designed with a planning system (Precision, ver. 3.3.1.3, Accuray), delivering a total dose of 42 Gy in six sessions (7 Gy per session), once weekly. This protocol was applied with palliative intent, aiming to achieve local tumor control and maintain quality of life, rather than curative eradication. The gross tumor volume (GTV) was defined based on the CT findings, and a planning target volume (PTV) was defined as the GTV plus a 3 mm margin. The prescribed dose aimed to ensure that 95% of the PTV received the total dose of 42 Gy. For immobilization, a customized dental mold was used as a bite block to maintain a reproducible position during each treatment session. Anesthesia was induced with intravenous administration of medetomidine, butorphanol, and midazolam. In the treatment planning phase, dose constraints were applied to the eyes, while the brain was not considered an organ at risk because of its distance from the target volume. Specific dose constraints were not applied to the oral mucosa. A dose–volume histogram (DVH) was generated to evaluate the treatment plan. A DVH was generated to evaluate the treatment plan (Figure 2). The GTV and PTV received adequate coverage with a total prescribed dose of 42 Gy in six fractions, while the mean doses to the right and left eyes were 5.67 and 8.52 Gy, respectively, which were considered acceptable. Informed consent was obtained from the pet owner.

**Figure 3:**
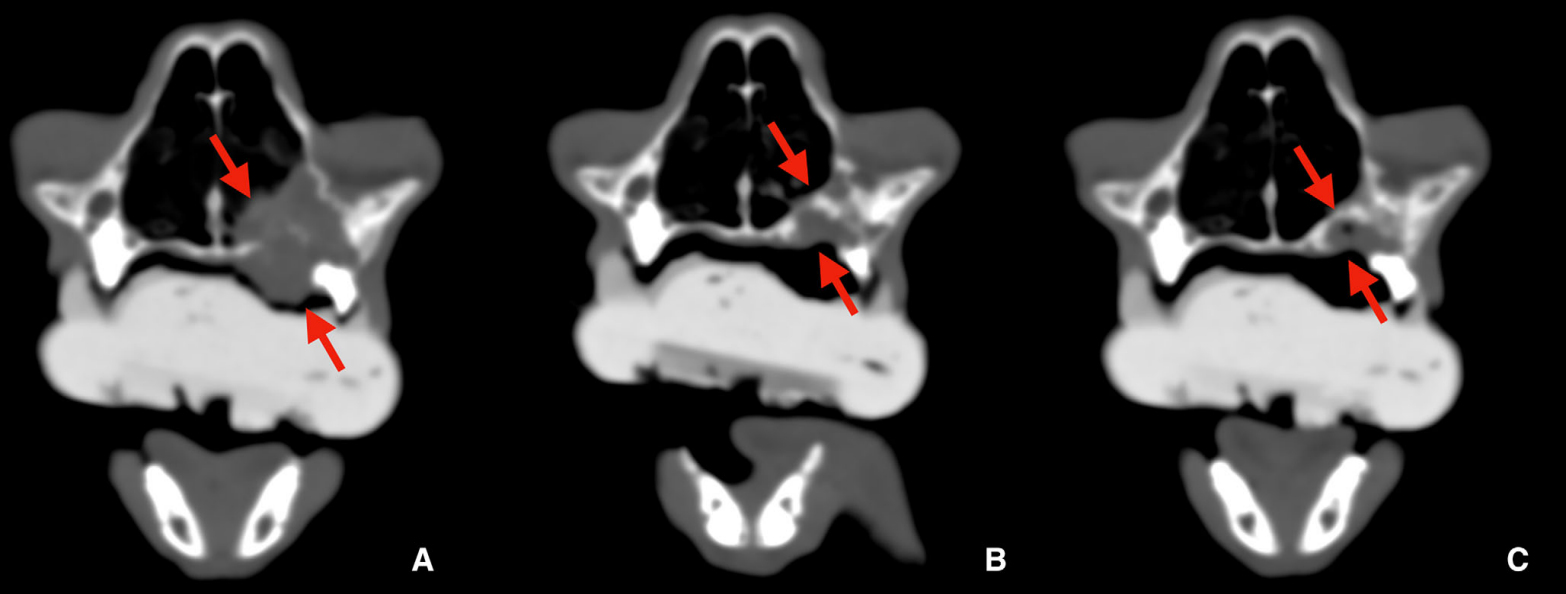
CT images of the same cat shown in bone window settings. (A) Pretreatment, (B) 2 months posttreatment, (C) 9 months posttreatment. The lesion is indicated with red arrows.

Radiation therapy was completed, and follow‐up CT scans were scheduled at 2 and 9 months posttreatment to assess the tumor's response. At the third treatment session, the cat's appetite showed slight improvement, though no change in tumor size was observed. By the fourth session, a noticeable reduction in the tumor size was evident. Two months after completion of therapy, a clinical remission was observed, with CT scans showing a reduction in tumor size (1.1 cm). Nine months posttherapy, the tumor had completely resolved (Figures 1 and 3). The cat remained in remission 481 days posttreatment (day 395), with no recurrence of the mass. The only recorded side effect was mucositis (Grade 2 VRTOG), but no significant radiation‐related injuries were observed, and the treatment was well tolerated.

## Discussion

Although surgical resection is considered the first‐choice treatment for APOT, due to its high local invasiveness, complete excision may be difficult depending on the tumor's location and size, and there is a high risk of local recurrence.^1^ In this case, the owner chose radiation therapy, taking these factors into consideration.

In dogs, previous reports described two APOT cases treated with radiation therapy, using 36 Gy/6 fractions (twice weekly) and 40 Gy/10 fractions (twice weekly). Both cases showed only short‐term tumor control (4 and 11 months, respectively). In contrast, the cat in the present study received 42 Gy/6 fractions (once weekly), resulting in complete remission and long‐term local control.^2^ Additionally, similar conditions in cats, such as feline inductive odontogenic tumor (FIOT) and multiple epulides, have been reported. FIOT is a benign tumor that occurs in young cats, with surgical resection being the primary treatment. One study reported that three cats underwent partial maxillectomy and showed no recurrence after long‐term follow‐up (3–5 years).^3^ Furthermore, a study of multiple epulides in cats reported that 13 cats underwent surgical resection, with 8 showing recurrence, but no signs of metastasis were observed.^4^ Additionally, in a study involving three cats with incomplete resection of odontogenic tumors (FIOT, odontogenic epithelial calcifying tumors, and epulides), radiation therapy as adjunctive treatment led to long‐term local control (over 35 months), with minimal radiation‐induced damage.^5^ These findings suggest that radiation therapy may be effective for odontogenic tumors, but the effects of radiation therapy alone are not clear. Thus, the role of radiation therapy in these similar conditions remains largely uncertain.

In this report, tumor shrinkage was first noted at the fourth radiation treatment. Complete remission was observed by day 133, 2 months after the conclusion of radiation therapy. No recurrence was detected during the 481 days follow‐up period, which suggests that radiation therapy may be an effective treatment for APOT in cats. This outcome indicates that radiation therapy may serve as a useful alternative treatment when surgical resection is not feasible for APOT cases. Radiation therapy may also be beneficial as an adjunct after incomplete surgical resection.

## Conclusion

APOT in cats is a rare condition, and there are few established treatment options other than surgical excision. This case demonstrates that radiation therapy may be an effective treatment for APOT in cats, with favorable outcomes and minimal radiation‐related side effects. Further studies are needed to define the optimal radiation therapy protocol and long‐term prognosis for APOT in cats.

## Conflicts of interest and sources of funding

The authors declare no conflicts of interest or sources of funding for the work presented here.

## Data Availability

The data that support the findings of this study are available from the corresponding author upon reasonable request.
